# Improving Diagnostic Robustness of Perfusion MRI in Brain Metastases: A Focus on 3D ROI Techniques and Automatic Thresholding

**DOI:** 10.3390/cancers17132085

**Published:** 2025-06-22

**Authors:** Stéphanie Rudzinska-Mistarz, Brieg Dissaux, Laurie Marchi, Anne-Charlotte Roux, Alexis Perrot, François Lucia, Romuald Seizeur, Olivier Pradier, Gurvan Dissaux, Moncef Morjani, Vincent Bourbonne

**Affiliations:** 1 Radiology Department, University Hospital, 29200 Brest, France; 2 Inserm, UMR 1304, GETBO, University of Western Brittany, 29238 Brest, France; 3 Inserm, UMR 1101, LaTIM, INSERM UMR 1101, University of Western Brittany, 29238 Brest, France; francois.lucia@chu-brest.fr (F.L.);; 4 Radiation Oncology Department, University Hospital, 29200 Brest, France; 5 Neurosurgery Department, University Hospital, 29200 Brest, France

**Keywords:** brain metastasis, stereotactic radiotherapy, radiation necrosis, perfusion MRI

## Abstract

**Background**: Distinguishing tumor recurrence from radiation necrosis after radiotherapy for brain metastases remains a major diagnostic challenge. Perfusion MRI, particularly the measurement of relative cerebral blood volume (rCBV), is a commonly used technique to differentiate between these two entities. However, variations in the placement of regions of interest (ROIs) affect diagnostic accuracy. This study compares the diagnostic performance of different cerebral perfusion methods, including a novel volumetric 3D ROI method and automatic thresholding, to differentiate tumor recurrence from radiation necrosis. **Methods**: We retrospectively analyzed data from 23 patients, including 25 brain metastases treated with stereotactic radiotherapy, who were suspected of local recurrence and had histological confirmation via biopsy or surgical resection. Each patient underwent perfusion MRI before surgery. The diagnostic performance of the different ROI methods (manual and 3D) was evaluated using the area under the ROC curve (AUC), as well as sensitivity and specificity measures. An automatic thresholding method was also applied, generating tumor sub-volumes with predefined cut-off values to determine the rCBV threshold most specific for differentiating relapse from necrosis. **Results**: The 3D ROI method, considering the whole lesion and a healthy ROI in the head of the caudate nucleus, demonstrated superior diagnostic performance (AUC = 0.65), outperforming manual methods (AUC = 0.53). Robustness was moderate, with an intraclass correlation coefficient of 0.60 between Syngo.via and IntelliSpace. **Conclusions**: The 3D ROI method shows promise in improving diagnostic accuracy in distinguishing tumor recurrence from radiation necrosis. Further studies with standardized protocols and larger populations are needed to validate these results.

## Simple Summary

Distinguishing between local relapse and damage from radiation-induced necrosis after brain radiotherapy remains a significant challenge. This research aims to improve the accuracy of diagnostic imaging, specifically perfusion MRI. The study evaluates new ways to analyze perfusion MRIs, integrating a technique that looks at the entire affected area in three dimensions and an automated method for setting measurement thresholds. By finding more reliable ways to interpret these images, the authors hope to help clinicians make more precise diagnoses. This could lead to better-tailored treatments for patients, preventing unnecessary or inappropriate therapies and ultimately improving patient outcomes.

## 1. Introduction

Radiation necrosis is a serious complication of radiotherapy that can arise months or even years after treatment, occurring in 5% to 25% of cases depending on the series. Local relapse refers to the reappearance or progression of a tumor following a period of remission. Radiologically, both conditions are characterized by new contrast enhancement or the progression of pre-existing enhancement in irradiated brain lesions. When these changes occur within the first six months after radiotherapy, they are typically considered pseudoprogression [1,2]. However, when they present later (from six months to several years), the diagnostic challenge between local relapse and radiation necrosis arises [1,3]. Local relapse is defined by tumor recurrence within the pre-treated volume. In contrast, radiation necrosis involves progressive tissue necrosis due to endothelial damage and demyelination, resulting in cell death and often lacking spontaneous resolution [4].

Distinguishing between these two conditions is crucial to orientate treatment strategies. Local relapse often requires an aggressive approach, such as surgery, systemic treatment, or additional radiotherapy, whereas radiation necrosis is generally managed with anti-inflammatory treatments like corticosteroids; anti-angiogenic treatments like bevacizumab [5]; and occasionally, surgical removal of necrotic tissue. Accurate diagnosis helps prevent unnecessary or inappropriate treatments, ultimately improving patient outcomes.

Differentiating between radiation necrosis and tumor recurrence, both clinically and radiologically, remains a significant challenge [6]. Various advanced imaging techniques have been investigated, including perfusion MRI, magnetic resonance spectroscopy (MRS) [7], and positron emission tomography (PET) [8], though none provide definitive diagnostic certainty. Histopathology remains the gold standard, although its invasive nature limits routine use.

Morphological MRI, currently the most commonly used modality, involves visual assessment of lesions through T1 and T2 sequences, along with post-contrast imaging to identify areas of abnormal enhancement. The pattern of contrast enhancement is also evaluated, and some studies have shown that “soap bubble” or “Swiss cheese” appearances are more frequently associated with radiation necrosis [9,10]. The performance of such patterns remains poor, with reported positive predicted value (PPV) rates of around 25% [11].

Perfusion MRI, particularly dynamic susceptibility contrast (DSC) sequences, has been reported as one of the most reliable methods for assessing cerebral perfusion [12]. Studies have shown that DSC-MRI can distinguish tumor recurrence from radiation necrosis with a sensitivity of 88% and a specificity of 75%, as evidenced by an area under the curve (AUC) of 0.84, leaving room for improvement of the diagnostic accuracy [13,14]. It measures parameters like relative cerebral blood volume (rCBV), a key marker of tissue vascularization. High rCBV suggests increased microvascular density [15], which is more commonly observed in tumor recurrence compared to radiation necrosis, due to tumor-associated neoangiogenesis. rCBV is calculated by placing regions of interest (ROIs) in the tumor and in healthy white matter [16].

However, this method has certain limitations due to factors such as the size and location of the ROIs, as well as the presence of necrosis or artifacts, which can introduce variability in the results [17]. Several studies have suggested improvements, such as using volumetric ROIs that exclude necrotic areas to reduce variability [18,19,20].

This study aims to compare various cerebral perfusion techniques to determine which method offers the most reliable diagnostic performance in distinguishing radiation necrosis from local relapse in patients with irradiated and subsequentially operated-on brain metastases.

## 2. Materials and Methods

### 2.1. Patient Selection

Patients were selected based on the following criteria: the presence of brain metastasis treated with stereotactic radiotherapy, suspicion of local relapse with histological confirmation by biopsy or surgery, available preoperative cerebral MRI with analyzable perfusion sequences, and a minimum lesion volume > 1 cc. This retrospective study was approved by the Ethics Committee of Brest University Hospital (29BRC23.0143), conducted in accordance with the Declaration of Helsinki, and patients were sent a non-opposition form for the use of their data. The study was registered on ClinicalTrials.gov (NCT06029140).

### 2.2. Dynamic Susceptibility Contrast (DSC) Sequences

Dynamic Susceptibility Contrast Perfusion-Weighted Imaging (DSC-PWI) relies on rapid acquisition of T2*-weighted sequences during the injection of a gadolinium-based contrast agent (DOTAREM or GADOVIST) to measure signal changes due to magnetic susceptibility effects as the contrast passes through cerebral vessels. Technical parameters include echo-planar imaging with short TR (1–2 s), TE around 30–50 ms, and slice thickness of 3–5 mm, for both 1.5 T and 3 T MRI. Perfusion maps, such as relative cerebral blood volume (rCBV), are computed using post-processing algorithms that involve deconvolution based on an arterial input function (AIF).

### 2.3. Processing of Cerebral Perfusion Maps

Cerebral perfusion data were processed using Syngovia VB60A_HF08 and IntelliSpace Portal 9.0 radiology software programs. Perfusion maps were automatically generated in Syngovia using the MR_Neurology viewer and the “Local AIF Perfusion Map” module, selecting the relRCBV corrected option. In IntelliSpace Portal 9.0, perfusion maps were generated using the MR_Neuro Perfusion_T2* mode, with rCBV corrected selected.

### 2.4. Lesion ROI Definition Delineation

Brain lesions were manually delineated using the MIM Maestro^®^ v7.1.3 radiotherapy software. The contours were drawn by tracing the outer edges of the lesion with the software’s 3D brush tool. The most commonly used conventional method involves placing a tumor ROI in the most enhanced part of the lesion, typically corresponding to the red zones on perfusion color maps, which indicate areas of high blood volume. This ROI aims to capture the hypervascularization within the tumor. Additionally, a reference ROI is placed in the contralateral “healthy” white matter, away from the lesion, serving as a baseline for normal perfusion. This is referred to as the healthy ROI, and its values are used to calculate ratios such as rCBV to distinguish pathological from normal tissue perfusion. Lesion ROIs were performed in 2 dimensions (2D) as per the reference method and in 3 dimensions (3D).

#### 2.4.1. Reference Method

Two senior neuroradiologists independently analyzed the cerebral perfusion using the standard method described above with the IntelliSpace Portal 9.0 software and subsequently reached a consensus on their values.

Apart from the reference, the delineation and placement of the different ROIs (lesion and healthy) were performed by an 8th-semester radiology resident under the supervision of a senior radiation oncologist and a neuroradiologist.

#### 2.4.2. Automatic Thresholding

**Figure 1:**
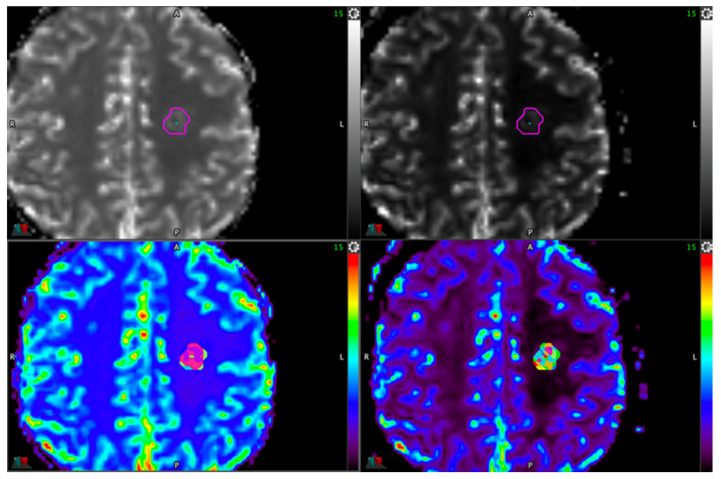
Automatic thresholding method using maps from Syngo.via (on the **left**) and IntelliSpace (on the **right**).

The automatic thresholding method involves several key steps. First, the delineation of the lesion is performed on the T1-weighted contrast-enhanced images using a detailed contouring method. Then, a registration process aligns the contrast-enhanced images with the perfusion sequence. Once aligned, the contour is transferred to the rCBV map, which was previously generated by either the Syngo.via v VB40A or IntelliSpace v8.0 software. Using the whole-tumor contour, several volumetric contours were automatically generated by MIM Maestro^®^ software, with predetermined cut-offs (none = all tumor, 5%, 10%, 20%, and so on up to 90%), creating multiple sub-volumes of the lesion using 95% of the maximal rCBV value as the reference. The mean pixel value was extracted, resulting in a mean rCBV value for each sub-volume. rCBV ratio values were then calculated by dividing the mean lesion rCBV value by the mean healthy ROI rCBV value. An example is provided in Figure 1.

#### 2.4.3. Healthy ROI Locations

To evaluate the impact of ROI location in the contralateral white matter on rCBV calculation, three locations were tested for the reference ROI: a homogeneous region in the contralateral white matter (CL) as described in the usual approach, the centrum semiovale (CSO), and the head of the caudate nucleus (NGC). Examples are shown as Supplementary Figures S1–S3. As for the lesion ROI, healthy ROIs were performed in 2D and 3D. Illustrative cases of local relapse and radiation necrosis are provided as Supplementary Figures S4 and S5.

### 2.5. Statistical Methods

The objective was to determine which methodology for the rCBV ratio (the definition of the lesion and placement of the healthy ROI) was the most accurate at distinguishing the local relapse from radiation necrosis. Combining the reference (1 value), the manual approach (3 healthy ROI placements × 2 software programs = 6 values) and the automatic approach (3 healthy ROI placements × 10 lesion volumes × 2 software programs = 60 values), 124 rCBV ratios (2D and 3D) were available per lesion. For clarity, only the reference, manual, and 6 best-performing combinations (one combination for each software and healthy ROI placement) are presented.

Statistical methods were applied to assess the performance of rCBV measurement techniques. Receiver Operating Characteristic (ROC) curves were generated for each method. The rCBV ratio threshold value was set in order to maximize the Youden Index. The area under the curve (AUC) was used to quantify the overall diagnostic performance. A subgroup analysis was also performed to compare AUCs between supra-tentorial and sub-tentorial tumor locations. The ground truth, defined as local recurrence (primary endpoint), was used as the reference to evaluate the accuracy of each method in distinguishing recurrence from radiation necrosis. Finally, the robustness of the rCBV ratios depending on Healthy ROI Placement and Software was assessed using the Cronbach’s alpha and the intra-class correlation coefficient (ICC) comparing Syngo.via and ISP. All figures and graphs were created using the online platforms GraphPad and Canva, while the statistical analysis was performed using MedCalc 15.8.

## 3. Results

### 3.1. Population

Thirty-four patients met these criteria and were initially included in the study. Of these, 10 patients did not have cerebral perfusion imaging in their follow-up and were therefore excluded, and 1 had lesions measuring less than 1cc. The remaining 23 patients underwent perfusion MRI in the three months prior to surgery. This cohort consisted of 13 men and 10 women, with a mean age of 64.4 years (range 48–88 years); 25 lesions were included, of which 15 were cases of local relapse and 10 were cases of radiation necrosis. Most MRIs were performed on a 1.5 T Siemens (20/25, 80%), while only 5 MRIs (20%) were performed on a 3 T Philips. Relevant clinical data are available in Supplementary Table S1.

### 3.2. Overall Performance of the Methods

The performance of the consensus method based on the combined evaluation by two senior radiologists demonstrated an AUC of 0.53, serving as the reference point for evaluating other techniques. Manual methods on IntelliSpace showed varying results depending on the placement of the ROI: the placement in the contralateral white matter (ISP_CL_Manual) yielded an AUC of 0.62, in the centrum semiovale (ISP_CSO_Manual) an AUC of 0.55, and in the head of the caudate nucleus (ISP_NGC_Manual) an AUC of 0.57. Similarly, manual methods on Syngovia showed comparable results: ROI placement in the contralateral white matter (Syngo_CL_Manual) had an AUC of 0.55, in the centrum semiovale (Syngo_CSO_Manual) an AUC of 0.53, and in the head of the caudate nucleus (Syngo_NGC_Manual) an AUC of 0.59. The automatic thresholding method demonstrated the best performance, with an AUC of 0.65 obtained using three different methodologies:

- IntelliSpace: whole tumor ROI + healthy ROI located the head of the caudate nucleus (All_ISP_NGC) and 5% tumor ROI + healthy ROI located in the centrum semiovale (5%_ISP_CSO)
- Syngo.via: whole tumor ROI + healthy ROI located the head of the caudate nucleus (All_Syngo_NGC).

None of the 2D-based rCBV ratios outperformed the corresponding 3D-based rCBV ratios.

### 3.3. Sensitivity and Specificity Analysis

Each method was evaluated in terms of sensitivity and specificity at various rCBV thresholds to determine its optimal diagnostic performance. The results show that certain methods, such as the manual methods with a healthy ROI in the contralateral white matter (ISP_CL_Manual and Syngo_CL_Manual), maximized specificity, reaching up to 100% for ISP_CL_Manual but with relatively low sensitivity (26.7% and 46.7%, respectively). In contrast, the automatic thresholding approach demonstrated a better balance. For instance, the 5%_ISP_CSO was associated with 60.0% sensitivity and 80.0% specificity.

Full results are provided as Table 1.

### 3.4. Impact of Automatic Thresholding on Mean rCBV Values

**Figure 2:**
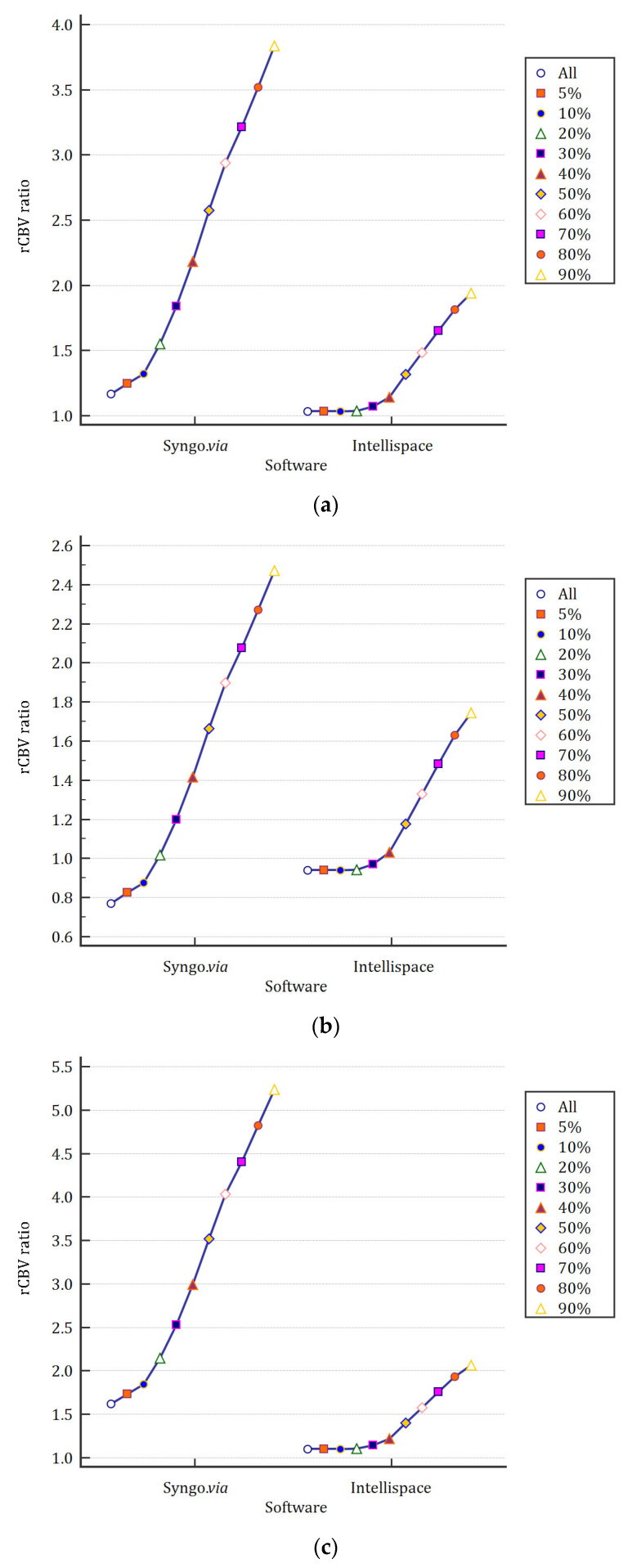
Mean rCBV values for each threshold depending on the software and the placement of the healthy ROI (CL, NGC, and CSO, respectively). (**a**) Healthy ROI placed in the contralateral white matter (CL). (**b**) Healthy ROI placed in the caudate nucleus (NGS). (**c**) Healthy ROI placed in the centrum semi-ovale (CSO).

Mean rCBV values for each threshold were plotted depending on the software. Figure 2a–c present the main results depending on the placement of the healthy ROI (CL, NGC, and CSO, respectively).

### 3.5. Robustness of rCBV Ratios Depending on Healthy ROI Placement and Software

For automatic volumes, our analysis demonstrates that the reference ROI placed in the head of the caudate nucleus (NGC) yields the highest concordance, with a non-standardized Cronbach’s alpha of 0.62, outperforming the centrum semiovale (CSO) and contralateral white matter (CL), which achieved values of 0.36 and 0.49, respectively (Table 2). This suggests that the head of the caudate nucleus provides greater reliability for rCBV measurements, particularly when using the whole tumor volume. However, for manually defined volumes, the CSO achieved the highest concordance.

### 3.6. Tumor Location Analysis

To further evaluate the performance of the different methods, we analyzed specific subgroups based on tumor location: infratentorial and supratentorial lesions. Our population includes six infra-tentorial brain lesions and 19 supra-tentorial lesions. For infra-tentorial tumors, the recurrence and necrosis rates were 66.70% and 33.3%, respectively. For supra-tentorial lesions, the recurrence rate was 57.9%, while necrosis occurred in 42.1% of cases. For infratentorial lesions, the manually defined rCBV ratios harbored higher AUCs than the majority of automatically defined values except Syngo_NGC_All. Detailed results are provided in Table 3.

## 4. Discussion

The results of our study demonstrate that the method using the healthy ROI placed in the head of the NGC and the whole 3D lesion offers the best diagnostic performance for distinguishing radiation necrosis from tumor recurrence, with an AUC of 0.65. This method outperformed traditional manual methods using the ROI in the contralateral white matter or the centrum semiovale. Manual methods showed lower performance, with AUCs of 0.41 for ISP_CL_Manual and 0.44 for Syngo_CL_Manual, and a moderately better performance for ROI placement in the head of the caudate nucleus, with an AUC of 0.58.

Our study highlights the significant advantages of using 3D ROI methods over traditional 2D ROI approaches, which often fail to capture the complex heterogeneity of tumor perfusion. By incorporating the entire lesion in 3D, this method provides a more comprehensive representation of the lesion’s vascular characteristics. This could explain the observed improvement in diagnostic accuracy. Previous studies, including those by Hu et al. [7] and Law et al. [21], have similarly shown that volumetric ROI methods enhance diagnostic performance by integrating spatial perfusion variations, which are not fully captured by conventional point-based measurements. 2D ROI can be drawn on a single slice either freeform to encompass the entire enhancing lesion or a smaller, fixed-diameter circular ROI specifically targeting the most abnormal area. This method is often favored for its simplicity and speed. In contrast, 3D ROI methodologies aim to encompass the entire volume of the lesion and can be time-consuming. While the vast majority of studies rely on 2D ROIs [16], it does not capture intratumoral heterogeneity and is prone to higher interobserver variability because of the choice of the tumoral ROI location.

Of note, the performance of the reference method used in our study was notably lower than anticipated, with an AUC of 0.53. This is significantly below the values reported in the literature. For example, in the meta-analysis by Teunissen et al. [22], the DSC perfusion method demonstrated an AUC of 0.83, with sensitivities and specificities reaching 0.89 and 0.78, respectively. The discrepancies between our findings and those in the literature may be attributed to several factors, including differences in cohort sizes, imaging protocols, and sampling biases. Large multicenter studies, such as those highlighted in the meta-analysis by Teunissen et al., rarely rely on histopathological confirmation, which may introduce both selection and classification bias and overestimate diagnostic performance. In contrast, our cohort includes a wider range of lesion progression statuses, reflecting a more realistic diagnostic challenge encountered in everyday clinical practice. While the meta-analysis by Teunissen et al. reported relatively high pooled sensitivities and specificities for the different advanced modalities, it outlined the considerable heterogeneity of reported values (e.g., I^2^ values ranging from 98.9 to 99.6 for advanced techniques) and the lack of universally accepted, externally validated diagnostic cut-off values. Of note, Teunissen et al.’s own external validation of DSC-rCBV thresholds demonstrated that sensitivities in an independent cohort were often lower (ranging from 0.47 to 0.77) than the reported pooled sensitivity (0.83), leading to a risk of misclassification. This observation underscores a critical challenge in the generalizability and reliability of current “gold standard” techniques, often stemming from variability in measurement protocols and the absence of standardized quantitative metrics.

In the existing literature, methods based on placing the ROI in the contralateral white matter are widely recommended for rCBV measurement due to the relative homogeneity of healthy white matter. However, contrary to previous reports, our results indicate that this practice demonstrated lower performance in our cohort. One potential explanation for this discrepancy could be the inclusion of patients with infratentorial lesions, where susceptibility artifacts and perfusion variations may render white matter ROI placement less reliable. On the other hand, ROI placement in the head of the caudate nucleus, though less conventional, showed slightly better performance. As suggested by Boxerman et al. [23], this region may be less prone to perfusion variations or motion artifacts, making it a more stable reference point.

Our results show slightly lower but consistent reliability for ROI placement in the centrum semiovale (CSO) for rCBV measurements compared to previous studies that often favor controlateral white matter. While our reliability indices are below commonly reported values (>0.80) [24], this may be due to differences in patient populations or imaging protocols. Improving ROI placement methods, such as the 3D approach, could enhance diagnostic performance.

Several confounding factors must also be considered when interpreting our results. The presence of necrotic areas within the tumor can lower rCBV values and mislead clinical interpretation, but the automatic thresholding method aims to reduce this confounding factor. The fact that retained volumes were all GTV or 5% GTV highlights the importance of considering the whole GTV. Thresholding did not improve the results—this approach being limited by the spatial resolution of perfusion maps. Additionally, the type of MRI machine (1.5 T vs. 3 T) can impact image quality, with higher field strengths leading to increased susceptibility artifacts [25]. This parameter was not available in our cohort due to the imbalance between the 2 sub-groups. Variability in post-processing software and differences in slice thickness further contribute to potential measurement discrepancies [26]. While the 3D ROI method offers a more systematic and comprehensive approach to lesion contouring, it is not immune to these limitations, and future studies should aim to standardize these parameters to ensure more reliable results. The authors of [27,28] have shown that the use of standardized protocols and advanced imaging techniques can significantly improve the reproducibility of measurements, which could explain the better performance of the 3D ROI method in our study.

There are several limitations to our study that must be acknowledged. First, the sample size and the predominance of supratentorial lesions in our cohort may limit the generalizability of our findings. The anatomical and vascular differences between infratentorial and supratentorial regions could affect rCBV values, making it difficult to directly compare our results with those of other studies [22]. While the limited number of cases included in our analysis as well as the retrospective setting were inherent to the study design, these points should be highlighted as limitations. We must point out that the selection of histologically confirmed cases only was made so that measurements could be compared to a robust gold standard. Errors in ROI placement, the quality of MRI images, and the application of processing algorithms were carefully reviewed and monitored to avoid measurement bias. While the 3D ROI methods offer a better representation of the tumor, they also require advanced skills for precise lesion contouring, which could introduce variations if these skills are not uniformly present among all radiologists. However, it is likely that the delineation of the entire lesion will more easily reach a consensus compared to the manual placement of ROIs.

To further validate the findings of this study, we recommend conducting a multicenter trial using standardized MRI acquisition protocols and ROI placement techniques. Such a study would help reduce inter-center variability and provide a clearer understanding of the true diagnostic performance of 3D ROI methods across different clinical settings. Based on our analysis, considering the whole GTV and healthy ROI placed in the head of caudate nucleus, using either Syngo.via or IntelliSpace will provide the best results.

## 5. Conclusions

In a cohort where the gold standard relies on histopathological confirmation, thus limiting the bias of misclassification, our study demonstrates the possible superiority of a new semi-automatic method for RCBV measurement. This method outperformed the reference method for the diagnosis of local relapse after stereotactic radiotherapy. Further validation is required and is currently ongoing in a non-operated cohort.

## Figures and Tables

**Table 1:** Performances of each lesion and healthy ROI combination depending on the software.

Sotware	Lesion ROI Definition	Healthy ROI Definition	Combination Denomination	AUC	*p*	rCBV Ratio Threshold	Se	Sp	PPV	NPV
ISP	Manual	CL	Reference	0.53	0.83	>1.65	33.33	90.00	83.33	47.37
ISP	Manual	CL	ISP_CL_Manual	0.62	0.31	≤0.94	26.67	100.00	100.00	47.62
		NGC	ISP_NGC_Manual	0.57	0.54	>1.39	33.33	90.00	83.33	47.37
		CSO	ISP_CSO_Manual	0.55	0.71	>2.14	53.33	70.00	72.73	50.00
	Automatic	CL	ISP_CL_5	0.57	0.60	≤1.03	60.00	60.00	69.23	50.00
		NGC	ISP_NGC_All	0.65	0.21	>1.01	40.00	90.00	85.71	50.00
		CSO	ISP_CSO_5	0.65	0.20	>1.12	60.00	80.00	81.81	57.14
Syngo	Manual	CL	Syngo_CL_Manual	0.55	0.70	≤1.42	46.67	70.00	70.00	46.67
		NGC	Syngo_NGC_Manual	0.59	0.47	>1.61	46.67	80.00	77.78	50.00
		CSO	Syngo_CSO_Manual	0.53	0.83	≤1.20	26.67	90.00	80.00	45.00
	Automatic	CL	Syngo_CL_5	0.53	0.84	≤1.21	46.67	30.00	50.00	27.27
		NGC	Syngo_NGC_All	0.65	0.18	>0.75	53.33	80.00	80.00	53.33
		CSO	Syngo_CSO_5	0.61	0.38	>1.88	46,67	80.00	77.78	50.00

Abbreviations: ISP: IntelliSpace; Syngo: Syngo.via; ROI: Region Of Interest; AUC: Area Under the Curve; rCBV: relative cerebral blood volume; Se: sensitivity; Sp: specificity; PPV: positive predictive value; NPV: negative predictive value; NGC: head of the caudate nucleus; CSO: centrum semiovale; CL: contralateral white matter; and combination denomination (*x*_*y*_*z*): x = software used for perfusion map generation, y = definition of the healthy ROI, and z = definition of the lesion ROI.

**Table 2:** Measures of interrater reliability on rCBV according to ROI locations.

Lesion ROI Definition	Healthy ROI Definition	Combination Denomination	Cronbach’s alpha		ICC (CI95%)	
			Raw	Standardized	Single Measures	Average Measures
Manual	CL	CL_Manual	0.56	0.57	0.39 (0.01–0.68)	0.56 (0.01–0.81)
	NGC	NGC_Manual	0.62	0.62	0.45 (0.08–0.72)	0.62 (0.15–0.83)
	CSO	CSO_Manual	0.68	0.69	0.52 (0.16–0.75)	0.68 (0.28–0.86)
Automatic	CL	CL_5	0.47	0.93	0.31 (−0.09–0.62)	0.47 (−0.20–0.77)
	NGC	NGC_All	0.60	0.92	0.43 (0.05–0.70)	0.60 (0.10–0.83)
	CSO	CSO_5	0.39	0.91	0.24 (−0.16–0.57)	0.39 (−0.39–0.73)

Abbreviations: ISP: IntelliSpace; Syngo: Syngo.via; ROI: Region Of Interest; ICC: Intraclass correlation coefficient; NGC: head of the caudate nucleus; CSO: centrum semiovale; CL: contralateral white matter; and combination denomination (*x*_*y*_*z*): x = software used for perfusion map generation, y = definition of the healthy ROI, and z = definition of the lesion ROI, CI95%: 95% confidence interval.

**Table 3:** Comparison of AUC of sub-tentorial vs. supra-tentorial lesions.

Software	Lesion ROI Definition	Healthy ROI Definition	Combination Denomination	Supra-Tentorial Lesion	Sub-Tentorial Lesion
				AUC (CI95%)	
ISP	Manual	CL	Reference	0.52 (0.29–0.75)	0.63 (0.19–0.94)
ISP	Manual	CL	ISP_CL_Manual	0.68 (0.43–0.87)	0.50 (0.12–0.88)
		NGC	ISP_NGC_Manual	0.52 (0.29–0.75)	0.75 (0.29–0.98)
		CSO	ISP_CSO_Manual	0.51 (0.28–0.74)	0.75 (0.29–0.98)
	Automatic	CL	ISP_CL_5	0.64 (0.39–0.84)	0.50 (0.12–0.88)
		NGC	ISP_NGC_All	0.68 (0.43–0.87)	0.50 (0.12–0.88)
		CSO	ISP_CSO_5	0.65 (0.40–0.85)	0.50 (0.12–0.88)
Syngo	Manual	CL	Syngo_CL_Manual	0.50 (0.27–0.73)	0.75 (0.29–0.98)
		NGC	Syngo_NGC_Manual	0.56 (0.32–0.78)	0.75 (0.29–0.98)
		CSO	Syngo_CSO_Manual	0.50 (0.27–0.73)	0.75 (0.29–0.98)
	Automatic	CL	Syngo_CL_5	0.57 (0.33–0.79)	0.63 (0.19–0.94)
		NGC	Syngo_NGC_All	0.64 (0.39–0.84)	0.75 (0.29–0.98)
		CSO	Syngo_CSO_5	0.59 (0.35–0.81)	0.63 (0.19–0.94)

Abbreviations: ISP: IntelliSpace; Syngo: Syngo.via; ROI: Region Of Interest; ICC: Intraclass correlation coefficient; NGC: head of the caudate nucleus; CSO: centrum semiovale; CL: contralateral white matter; combination denomination (*x*_*y*_*z*): x = software used for perfusion map generation, y = definition of the healthy ROI, and z = definition of the lesion ROI; AUC: Area Under the Curve; and CI95%: 95% confidence interval.

## Data Availability

The original contributions presented in this study are included in the article/Supplementary Materials. Further inquiries can be directed to the corresponding author.

## Supplementary Materials

The following supporting information can be downloaded at https://www.mdpi.com/article/10.3390/cancers17132085/s1, Table S1. Details of clinical data of each case. Figure S1. Reference ROI in the controlateral white matter. Figure S2. Reference ROI in the centrum semiovale. Figure S3. Reference ROI in head of the caudate nucleus. Figure S4. Illustration of a case of local relapse. Figure S5. Illustration of a case of radiation necrosis.
