# Supplementary material for: Improving Diagnostic Robustness of Perfusion MRI in Brain Metastases: A Focus on 3D ROI Techniques and Automatic Thresholding
Source: Cancers (Basel). 2025 Jun 22;17(13):2085. doi: 10.3390/cancers17132085 (PMC12249341; doi:10.3390/cancers17132085)

SUPPLEMENTARY MATERIALS

Supplementary Table S1: Details of clinical data of each case
Supplementary Figure S1: Reference ROI in the controlateral white matter
Supplementary Figure S2 Reference ROI in the centrum semiovale
Supplementary Figure S3 Reference ROI in head of the caudate nucleus
Supplementary Figure S4: Illustration of a case of local relapse
Supplementary Figure S5: Illustration of a case of radiation necrosis

Supplementary Table S1: Details of clinical data of each case

Histology	Location	Laterality	Volume	Time from SRT	Symptoms	Edema	Corticosteroids	Bevacizumab	Local Relapse
Triple negative breast cancer	Brainstem	N/A	4.87	3,15	Yes	Yes	Yes	No	Yes
HER2+ breast cancer	Frontal	Left	1.26	5,80	No	Yes	No	No	No
Small cell lung cancer	Parietal	Left	29.02	10,43	No	Yes	No	No	No
Non-small cell lung cancer (adenocarcinoma)	Parietal	Right	31.17	46,62	No	No	No	No	No
Melanoma	Frontal	Left	3.07	25,11	No	Yes	No	No	No
Non-small cell lung cancer (adenocarcinoma)	Frontal	Left	3.48	11,11	No	Yes	No	No	No
HER2+ breast cancer	Cerebellum	Right	2.77	30,66	No	Yes	No	No	Yes
Non-small cell lung cancer (adenocarcinoma)	Frontal	Right	1.97	7,61	No	Yes	No	No	Yes
Non-small cell lung cancer (adenocarcinoma)	Cerebellum	Right	14.04	15,15	Yes	Yes	Yes	No	No
Small cell lung cancer	Cerebellum	Left	50.18	15,44	No	Yes	No	No	Yes
Melanoma	Occipital	Right	22.96	1,51	Yes	Yes	Yes	No	Yes
Non-small cell lung cancer (adenocarcinoma)	Parietal	Left	5.81	11,11	No	Yes	No	No	Yes
Melanoma	Cerebellum	Right	11.46	1,41	Yes	Yes	Yes	No	Yes
HER2+ breast cancer	Cerebellum	Right	0.94	28,52	Yes	Yes	Yes	No	No
HER2+ breast cancer	Cerebellum	Right	4.87	40,92	Yes	Yes	Yes	No	Yes
Melanoma	Frontal	Left	1.24	14,46	No	Yes	No	No	Yes
Renal cell carcinoma	Cerebellum	Right	6.55	57,84	No	Yes	No	No	Yes
Small cell lung cancer	Occipital	Left	11.73	6,20	Yes	Yes	Yes	No	No
Non-small cell lung cancer (adenocarcinoma)	Parietal	Left	0.88	6,46	No	Yes	No	No	Yes
Non-small cell lung cancer (adenocarcinoma)	Parietal	Left	1.05	10,59	Yes	Yes	Yes	No	No
Non-small cell lung cancer (adenocarcinoma)	Parietal	Left	9.61	18,72	Yes	Yes	Yes	No	No
Non-small cell lung cancer (Squamous)	Parietal	Left	2.29	23,77	Yes	Yes	Yes	No	No
Melanoma	Frontal	Right	13.03	18,23	No	Yes	No	No	Yes
Non-small cell lung cancer (Squamous)	Frontal	Left	1.76	8,00	Yes	Yes	Yes	No	Yes
Melanoma	Frontal	Left	3.78	16,92	Yes	Yes	Yes	No	Yes

Non-small cell lung cancer (adenocarcinoma)	Temporo-occipital	Right	4.55	28,95	No	Yes	No	No	Yes
Triple negative breast cancer	Parieto-occipitatl	Left	11.4	8,82	No	Yes	No	No	Yes

Abreviations : SRT : Stereotactic Radiotherapy

Supplementary Figure S1: Reference ROI in the controlateral white matter

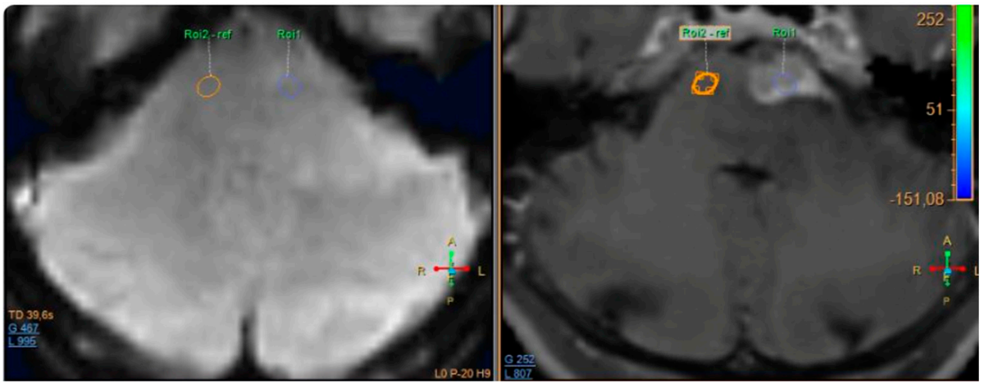

Supplementary Figure S2 Reference ROI in the centrum semiovale

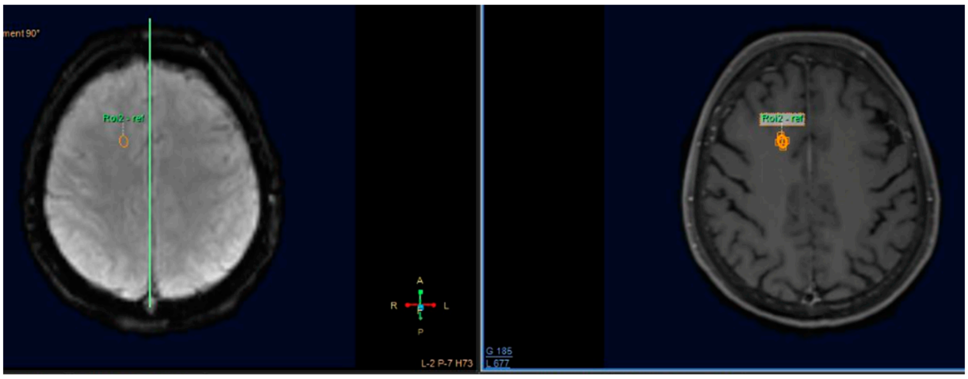

Supplementary Figure S3 Reference ROI in head of the caudate nucleus

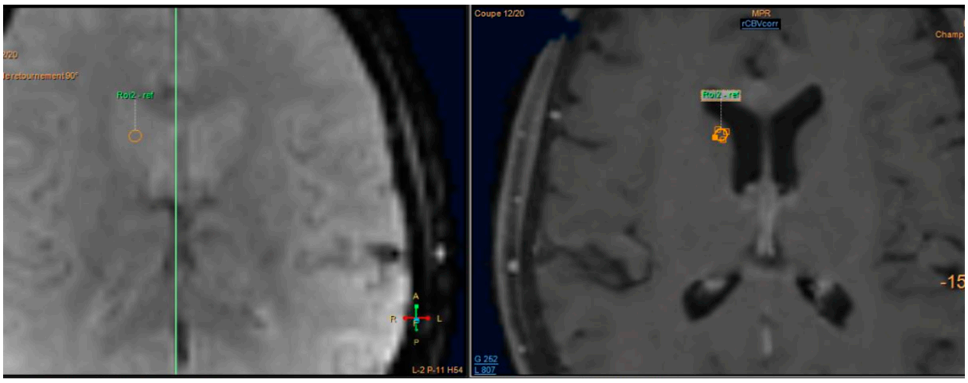

Supplementary Figure S4: Illustration of a case of local relapse

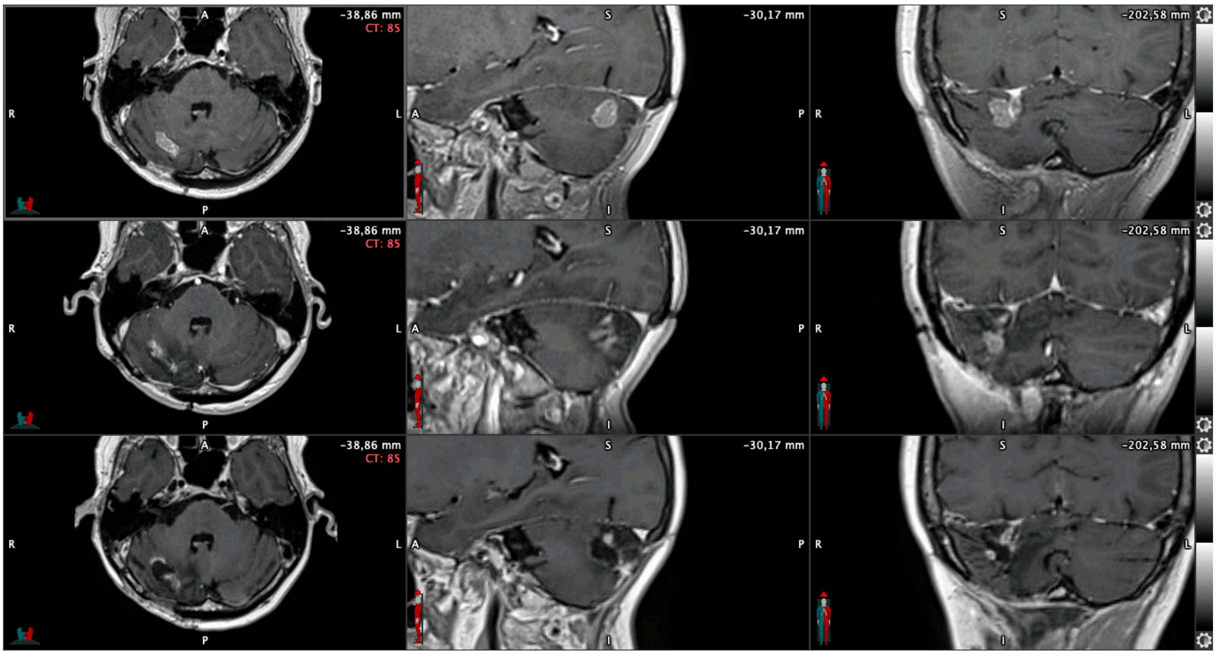

Caption: Top = Before radiotherapy, middle = before surgery, bottom = after surgery (confirmation of a local relapse)

Supplementary Figure S5: Illustration of a case of radiation necrosis

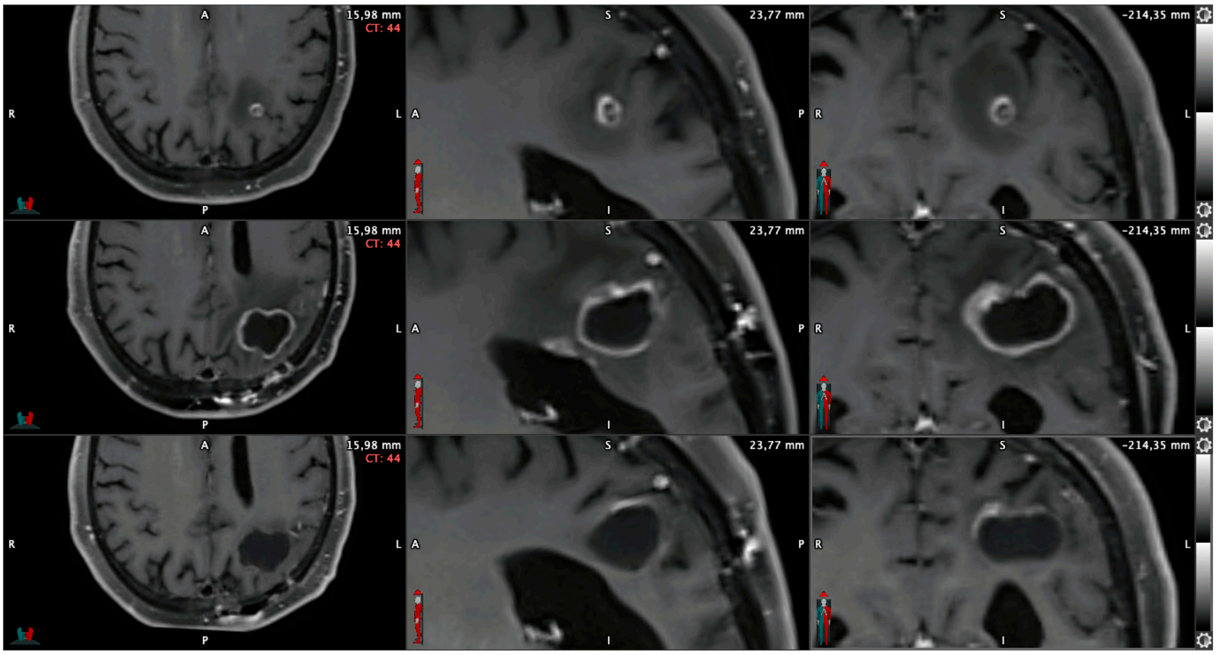

Supplement: Supplementary file 1 [file cancers-17-02085-s001.zip › cancers-3690080-supplementary.pdf]
